# Centrofacial cutaneous and oral ulcerations associated with pansinusitis

**DOI:** 10.1016/j.jdcr.2021.10.021

**Published:** 2021-11-18

**Authors:** Travis S. Dowdle, Jeannie M. Nguyen, Ashley L.E. Sturgeon, Michelle B. Tarbox, Cloyce L. Stetson

**Affiliations:** a School of Medicine, Texas Tech University Health Sciences Center, Lubbock, Texas; b Department of Dermatology, Texas Tech University Health Sciences Center, Lubbock, Texas

## Case presentation

**Figure 1:**
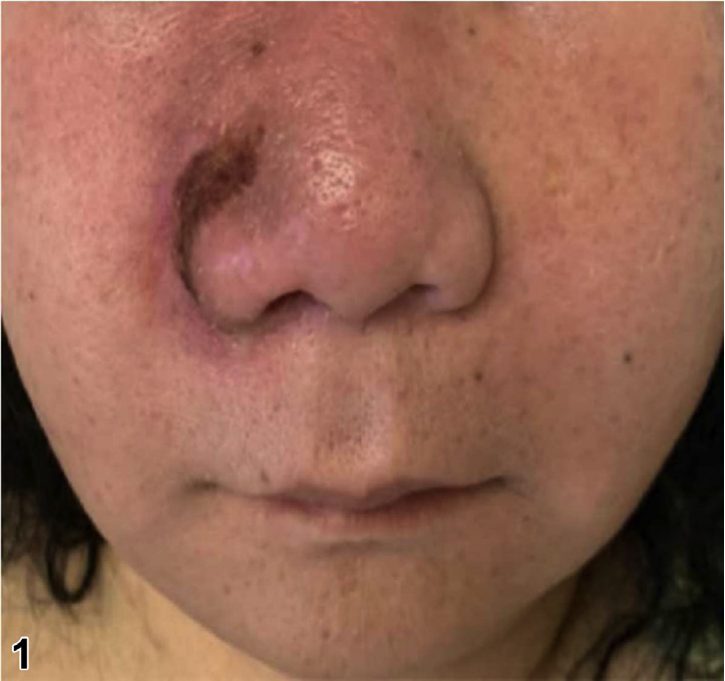
By Travis S. Dowdle, BS; Jeannie M. Nguyen, MD; Ashley E. Sturgeon, MD; Michelle B. Tarbox, MD; Cloyce L. Stetson, MD.

**Figure 2:**
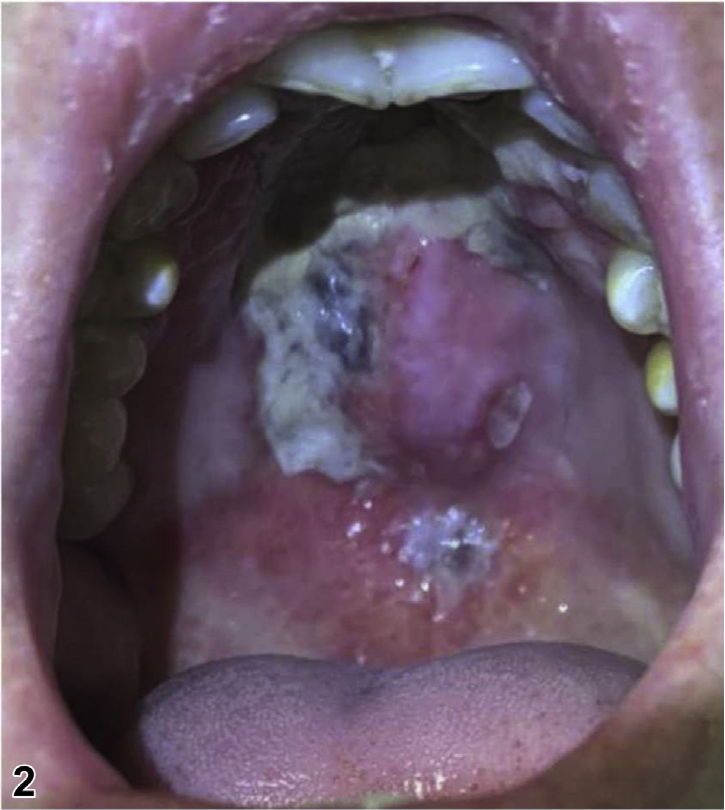
By Travis S. Dowdle, BS; Jeannie M. Nguyen, MD; Ashley E. Sturgeon, MD; Michelle B. Tarbox, MD; Cloyce L. Stetson, MD.

**Figure 3:**
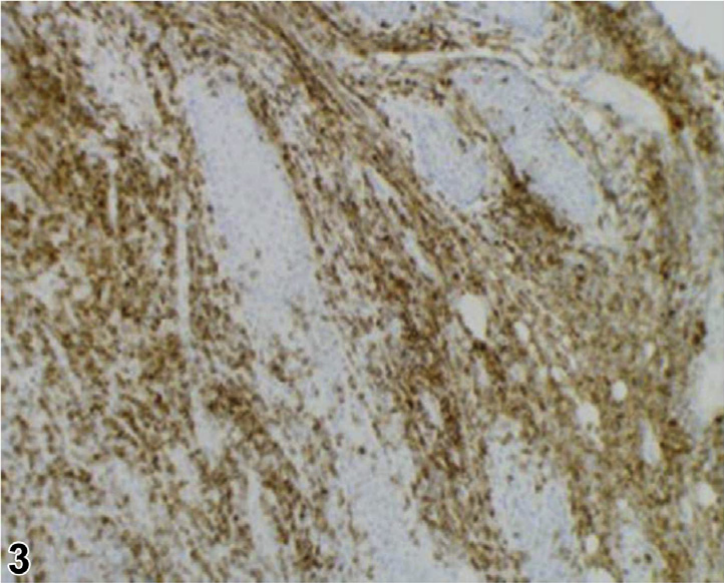
By Travis S. Dowdle, BS; Jeannie M. Nguyen, MD; Ashley E. Sturgeon, MD; Michelle B. Tarbox, MD; Cloyce L. Stetson, MD.

A 56-year-old Vietnamese woman presented with worsening chronic pansinusitis with progressive oral and cutaneous involvement. For the previous 8 months, she had undergone multiple endoscopic submucosal resections of the nasal turbinates. Despite debridement and many courses of oral and intravenous (IV) antibiotics, her clinical course worsened. While further tests were pending, dermatology was consulted. On the day of consultation, physical examination showed diffuse centrofacial edema, ulceration of the right alar groove (Fig 1), and necrosis of her soft palate (Fig 2). A punch biopsy was performed of the skin adjacent to the ulcer and the dermal infiltrate stained positively for CD56 (Fig 3, 100×).

**Question #1: What is the most likely diagnosis?**

A. Rhinosporidiosis
B. Nasal extranodal natural killer (NK)/T-cell lymphoma (ENKTL)
C. Granulomatosis with polyangiitis
D. Extranasal ENKTL
E. Paracoccidioidomycosis

Click here to view disclosures, take the quiz, and claim CME credit.

## Conflicts of interest

The authors have no conflicts of interest to declare.

